# International multicenter prospective evaluation of PAS management in 23 IS‐PAS member centers

**DOI:** 10.1111/aogs.15120

**Published:** 2025-04-11

**Authors:** Thorsten Braun, Sally Collins, Olivier Morel, Ammar Al Naimi, Charline Bertholdt, Albaro Jose Nieto‐Calvache, Frederic Chantraine, Alexander Paping, Vedran Stefanovic

**Affiliations:** ^1^ Department of Obstetrics, Charité – Universitätsmedizin Berlin Corporate Member of Freie Universität Berlin and Humboldt‐Universität zu Berlin Berlin Germany; ^2^ Division of ‘Experimental Obstetrics’ Charité – Universitätsmedizin Berlin, Corporate Member of Freie Universität Berlin and Humboldt‐Universität zu Berlin Berlin Germany; ^3^ Nuffield Department of Women's and Reproductive Health University of Oxford Oxford UK; ^4^ Birmingham Women's Hospital Birmingham UK; ^5^ Department of Obstetrics Nancy Regional and University Hospital Center (CHRU), Université de Lorraine Nancy France; ^6^ Department of Obstetrics and Gynecology, Division of Obstetrics & Maternal Fetal Medicine University Hospital Frankfurt Goethe‐University Frankfurt Germany; ^7^ Department of Obstetrics and Gynecology Buergerhospital Frankfurt Germany; ^8^ Unit of High Complexity Obstetrics, Gynecology and Obstetrics Department Fundación Valle del Lili Cali Colombia; ^9^ Faculty of Health Sciences Universidad ICESI Cali Colombia; ^10^ Department of Obstetrics and Gynecology CHU de Liège, Hospital Citadelle Liège Belgium; ^11^ Department of Obstetrics and Gynecology Fetomaternal Medical Center, Helsinki University Hospital and University of Helsinki Helsinki Finland

**Keywords:** abnormally invasive placenta, cesarean section, focal resection, complication, high‐risk pregnancy, hysterectomy, myometrial resection, postpartum hemorrhage

**Key message**

The International Society for Placenta Accreta Spectrum (IS‐PAS) aims to improve the diagnosis and management of PAS. This second analysis of our international database incorporates prospectively collected data from IS‐PAS member centers. Our online database has facilitated collaborative international multicenter observational studies of pregnancies with PAS, allowing us to analyze key clinical issues, identify areas of uncertainty, and propose new areas of PAS research. The findings highlight the need for rigorous prospective study designs to address specific research questions in the future.

The International Society for Placenta Accreta Spectrum (IS‐PAS) has transitioned from a small European Working Group (EW‐AIP) into an international interdisciplinary group of centers with expertise in placenta accreta spectrum (PAS) management. Such expertise typically encompasses maternal‐fetal medicine, gynecologic surgery, gynecologic oncology, vascular surgery, trauma surgery, urologic surgery, transfusion medicine, critical care specialists (intensivists), neonatologists, interventional radiologists, anesthesiologists, specialized nursing staff, and ancillary personnel, as described by Silver et al.^1^ Since its inception in 2017, IS‐PAS has grown to 71 active members from 47 centers across 27 countries with varying income levels.

IS‐PAS aims to generate high‐quality research on all aspects of PAS, including diagnostics and management, while enhancing education for healthcare professionals and patients. The organization has developed informative flyers in multiple languages to assist women in understanding PAS, available for free on its website (www.is‐pas.org). To become a registered center, applicants must demonstrate the management of at least ten PAS cases (Grades 2–3) over three years and submit relevant documentation, including data collection forms and intraoperative photographs. Once these criteria are met, the board reviews the application.

Affordable membership fees, with reduced rates for those from low‐income countries, primarily support administrative costs and database maintenance. In this supplement, we present a unique international multicenter prospective evaluation of PAS management. The web‐based database of pregnancies complicated by PAS has been completely revised and adapted to meet the current requirements and latest findings from the literature.

In 2021, IS‐PAS published a series of articles in *Acta Obstetricia et Gynecologica Scandinavica* focusing on the diagnosis and management of placenta accreta spectrum (PAS), based on a newly developed multicenter database.^2^, ^3^, ^4^, ^5^, ^6^, ^7^ The custom‐made, web‐based, secure online database FetView (FetView; Zeitgeist Health SE) was implemented in 2016 and can receive strictly anonymized woman‐related textual data and allows statistical queries. After the analysis of the first data set, numerous measures were taken to further improve the data quality. The new database incorporates both the IS‐PAS grading^8^ and the updated FIGO classification for intraoperative PAS assessment,^9^ allowing for direct comparisons between the systems. Possibilities of more detailed descriptions of antenatal ultrasound (US) or magnetic resonance imaging (MRI) markers were incorporated, and the full terminology of markers defined by IS‐PAS was embedded in the new query. Numerous query fields were made mandatory fields to obtain a complete data set with high quality.^10^ This enhanced database facilitated deeper analyses of PAS diagnosis and management, improving insights into risk factors and prevention strategies. All participating centers operated under local ethical approval and Data Use Agreements. Details of these have been published previously.^2^ We thank David Dostal, FetView, Zeitgeist Health SE, Prague, Czech Republic, for his great work in setting up and managing the database. We would like to thank all the members of the International Society for Placenta Accreta Spectrum for providing data from their PAS cases.

Between 2020 and 2022, 23 member centers from 16 countries representing high‐ and mid‐income countries from Europe, North‐ and South America contributed data of 315 PAS cases. All centers are specialized in PAS management.^11^, ^12^

This supplement provides some new insights into PAS grading accuracy, uterus‐conserving surgery applications, risk prediction models, and the associations of PAS with fetal anomalies, stillbirth, and neonatal morbidity, consisting of four papers with results from our database^13^, ^14^, ^15^, ^16^ and two papers whose at least one author is a member of IS‐PAS.^17^, ^18^

While the alignment of management strategies among centers has improved, most IS‐PAS participants come from well‐equipped hospitals in countries with well‐funded health systems, necessitating careful interpretation of the data concerning its generalizability. Given the correlation between PAS with birth rates and cesarean deliveries, countries experiencing high birth rates and frequent cesarean deliveries are more significantly impacted. Regions with limited resources, such as South America, North Africa, and parts of Asia, are witnessing a rise in cesarean delivery rates, highlighting the need for targeted research strategies in these areas.

This supplement benefits from the FIGO PAS classification introduced in 2019, addressing crucial research questions around PAS diagnosis and management. It addresses the predictive values of ultrasound and MRI, the risk factor assessment for delivery, the evaluation of uterus‐preserving PAS management, the definition of the optimal timing of delivery and related maternal and neonatal outcomes, and the evaluation of the new FIGO grading and the development of a severity PAS risk calculator. These comprehensive analyses reveal insights into management practices across different countries, underscoring limitations and opportunities for improvement that may not be evident from a single‐center perspective.

Appropriate management depends on clinical severity, individual patient preferences, and the expertise of the treating team. Our database has enabled us to compare various management strategies in the context of PAS management.^16^ Most IS‐PAS centers favor hysterectomy over conservative techniques. However, focal resection may be equally effective for women who wish to preserve their uterus. The acceptance of uterus‐preserving treatment strategies could be enhanced by developing objective criteria regarding when and how to implement these methods, as well as by providing systematic training (e.g., through the use of models) for PAS specialists.^16^

The effectiveness of the Sargent model^1^ in distinguishing between abnormally adherent placenta (FIGO grade 1) and abnormally invasive placenta (FIGO grades 2 and 3) has been evaluated.^14^ The study highlights the challenges associated with developing universally applicable prediction models for PAS. The findings emphasize the necessity for updating current ultrasound descriptors and for the development of new predictive models that can utilize data collected by various operators in multiple clinical environments. Although the Sargent model holds potential for clinical practice, it may present challenges in low‐ and middle‐income countries due to limited access to imaging technologies. Nevertheless, with its user‐friendly diagram format, the Sargent model remains a powerful tool in centers specialized in the management of PAS.^14^

One of our studies revealed that antenatal bleeding and the location of the placenta, distant from the uterine scar, are independent risk factors for emergent delivery among patients with PAS.^15^ However, emergency deliveries were not associated with severe adverse maternal outcomes, likely because these cases were managed in specialized centers that provide around‐the‐clock support. Certainly, this may not apply to centers with limited expertise in PAS management, underscoring the importance of antenatal screening for PAS and the timely referral of such cases.^15^

While most previous studies on PAS outcomes have primarily focused on maternal outcomes, there is limited data concerning fetal outcomes. A further manuscript evaluates the association between PAS and fetal malformations, stillbirth, neonatal death, and neonatal morbidity.^13^ The IS‐PAS database demonstrated that although PAS may increase the risk of some of these outcomes, the absolute rates of fetal and neonatal morbidity and mortality remain low and are primarily related to prematurity. Given the nature of the IS‐PAS database and the absence of a control group, this study is not a comparative analysis of relative risk; rather, it is a survey of the data we collected within our centers, highlighting the need for further prospective large‐scale studies in diverse settings.^13^

A systematic review by Adu‐Bredu et al. showed that the previously published scoring systems do not have clearly defined diagnostic criteria.^17^ While these scoring systems can effectively differentiate between scar dehiscence with an underlying non‐adherent placenta and high‐grade placental accreta spectrum (PAS) with excellent diagnostic accuracy, they are less effective for low‐grade PAS.^17^ Therefore, relying solely on scoring systems may result in errors in estimating the risk or extent of the condition, which can hinder appropriate preoperative planning.

The European Working Group for Abnormally Invasive Placenta (now IS‐PAS) previously proposed a checklist of ultrasound features for the antenatal detection of abnormal placentation conditions.^10^ The study conducted by Bartel et al. demonstrated that there is no single ultrasound feature that reliably predicts abnormal placentation; rather, it is the combination of features within the checklist that yields strong performance metrics.^18^ While many ultrasound features associated with abnormal placentation are also observed in cases of placenta previa following prior cesarean deliveries, we note that these cases typically do not exhibit multiple features simultaneously. Therefore, standardizing ultrasound assessments using this checklist proves beneficial for the prenatal detection of abnormal placentation.

The nature of previous IS‐PAS database studies has inherent limitations. Caution is needed when comparing clinical outcomes across facilities with different diagnostic and management protocols. While randomized controlled trials in a surgical context are challenging,^19^ previous research indicates their feasibility.^20^ Recognizing our current limitations, we must strive to standardize interventions and design prospective studies, including randomized trials, to expand treatment options for women with PAS in specialized centers.

PAS is a poorly understood condition, and recent analyses of experiences from various hospitals have enhanced our understanding of this rare condition, leading to a shift in concepts previously viewed as statuses. However, large cohort studies are still lacking, and a comprehensive understanding of PAS pathophysiology is needed to develop effective prevention strategies. Therefore, international multidisciplinary and multicenter academic organizations like IS‐PAS must tackle these issues. Significant changes in the definition, classification, diagnostic protocols, pregnancy management, and surgical treatment for PAS are anticipated in the coming years, driven by the needs of women and interdisciplinary groups. Moreover, high‐quality research with prospective designs might shed light on the underlying biological mechanisms contributing to the pathophysiology of PAS and must be prioritized to incorporate recent advancements in understanding the disease. Exploring genetic, epigenetic, and environmental factors could provide valuable information about individual susceptibility and progression of the condition. In summary, the future of studies concerning the biology of PAS appears promising, with advancements in diagnosis, treatment, and comprehensive care strategies on the horizon. These efforts will ultimately enhance our ability to manage this challenging condition effectively, improving outcomes for mothers and their infants.

## AUTHOR CONTRIBUTIONS

**Thorsten Braun**: Conceptualization, data curation, formal analysis, investigation, methodology, project administration, resources, visualization, writing—original draft. **Alexander Paping**: Conceptualization, data curation, formal analysis, investigation, methodology, project administration, resources, visualization. **Sally Collins, Olivier Morel, Ammar Al Naimi, Charline Bertholdt, Albaro Jose Nieto‐Calvache, Frederic Chantraine, and Vedran Stefanovic**: Resources, validation, writing—review and editing.
